# Assess the level of consciousness in patients with disorders of consciousness by combining resting-state and auditory-evoked EEG

**DOI:** 10.3389/fnins.2025.1613356

**Published:** 2025-11-11

**Authors:** Wenjin Zhang, Xiaochu Shi, Meng Li, Lipeng Zhang, Rui Zhang, Xing Wu, Mengjie Xin, Runtao Li, Hui Zhang, Yuxia Hu

**Affiliations:** 1 Central Hospital Affiliated to Zhengzhou University, Zhengzhou, China; 2 School of Electrical and Information Engineering, Zhengzhou University, Zhengzhou, China; 3 Henan Key Laboratory of Brain Science and Brain-Computer Interface Technology, Zhengzhou, China; 4 Institute of Neuroscience, Zhengzhou University, Zhengzhou, China

**Keywords:** electroencephalography, disorder of consciousness, resting-state, auditory-evoked potential, nonlinear dynamics feature, diagnostic

## Abstract

**Introduction:**

Electroencephalography (EEG) can provide objective neural marker for assessing the level of consciousness of patients with disorders of consciousness (DoC), but current research mainly focuses on the EEG features of a single modality, such as the resting-state or the evoked state, which results in less than ideal assessment accuracy. To accurately assess the level of consciousness of DoC patients, we proposed a new method by combine with resting-state and auditory-evoked EEG.

**Methods:**

The EEG data of resting-state and auditory-evoked potential were collected from 157 DoC patients. Then, nonlinear dynamics feature (NDF) include spatiotemporal correlation entropy and neuromodulation intensity of multimodal EEG were extracted. Next, the multi-form feature selection algorithm (MFFS) was adopted to optimize the extracted EEG features. Finally, a diagnosis model was constructed using support vector machine (SVM).

**Results:**

Among them, SC-Theta, SC-Alpha, NI-Alpha and ERP features were significantly (*p* < 0.05) correlated with the patient’s level of consciousness, resulting in an average grouping accuracy of 92.4%.

**Discussion:**

The proposed diagnostic model has demonstrated its distinctive advantages, showcasing remarkable effectiveness and reliability in accurately assessing consciousness states. This method holds promise for improving the reliability of clinical awareness assessments.

## 1 Introduction

Disorder of consciousness (DoC) is a type of brain function disorder caused by damage or dysfunction of the neural systems that regulate arousal and consciousness. It is typically classified into two levels: unresponsive wakefulness syndrome (UWS) and minimally conscious state (MCS) (Giacino et al., 2014; Edlow et al., 2021), UWS may show similar brain activity to MCS (Thibaut et al., 2021). Accurate diagnosis of the level of consciousness is of great importance for making treatment decisions for patients. Currently, clinical assessment of consciousness level is mainly based on the Coma Recovery Scale-Revised (CRS-R) behavioral assessment score sheet (Bruno et al., 2010). However, the assessment results are easily affected by the patient’s motor ability and the subjective will of the evaluating doctor, and there is still a high misdiagnosis rate (Song et al., 2020; Song et al., 2021). Therefore, there is an urgent need for more objective assessment methods to assist medical staff in completing the assessment work. Electroencephalography (EEG) can directly reflect the functional state of the brain and has advantages such as easy data acquisition and low cost. Currently, many researchers have used it in the study of consciousness disorders in patients (Bai et al., 2020; Formaggio et al., 2020; Fernandez-Espejo and Owen, 2013).

Resting-state EEG is commonly used in studies of consciousness assessment, with linear power spectrum analysis of five commonly used frequency bands including Delta (1 ~ 4 Hz), Theta (4 ~ 8 Hz), Alpha (8 ~ 13 Hz), Beta (13 ~ 30 Hz), and Gamma (30 ~ 45 Hz). In DOC patients, the energy of Delta and Theta bands is higher than in normal subjects (Piarulli et al., 2016; Naro et al., 2018), while the energy of Alpha band is lower (Chennu et al., 2016; Babiloni et al., 2009). However, studies on Beta and Gamma bands are still limited. Schiff’s “ABCD” model can explain the changes in EEG characteristics from coma to wakefulness (Schiff, 2022). In most cases, neural discharge in the brain is not continuous and fixed, but intermittent and brief (Feingold et al., 2015; Lundqvist et al., 2016). This leads to non-stationary and non-linear characteristics of the EEG signal. Thus, there may be some easily overlooked nonlinear features in the EEG signal, such as Spatiotemporal correlation entropy (Freeman and Quiroga, 2012) Neuromodulation Intensity(NI), neuromodulations plays an important role as a biomarker in the field of Neuroengineering and has repeatedly played a role in applications such as sleep analysis (Rechtschaffen, 1978), brain machine interface (Lebedev and Nicolelis, 2006), epilepsy research (Mormann et al., 2000), and neurorehabilitation (Daly and Wolpaw, 2008).

Auditory evoked EEG can reflect the preservation of the patient’s auditory nerve conduction pathway. The Oddball paradigm is currently the most commonly used paradigm for evoking auditory potentials (Perrin et al., 2006). This paradigm can evoke the N1 component, which characterizes the necessary auditory perception process, and the mismatch negativity (MMN) component, which characterizes the automatic deviation recognition process. For these two components, Kotchoubey et al. found that the proportion of N1 component in VS patients was significantly lower than that in MCS patients (Kotchoubey et al., 2005). Wijnen et al. found that the amplitude of MMN significantly increased with the recovery of consciousness level (Wijnen et al., 2007). Recent studies by Wang et al. also showed a significant correlation between MMN amplitude and the CRS-R score of DoC patients (Wang et al., 2018; Wang et al., 2020). Although the above studies suggest that N1 and MMN are correlated with the residual consciousness level of patients, it is still difficult to assess the patient’s consciousness level solely based on auditory-evoked EEG components.

In summary, current research on the assessment of consciousness levels mainly focuses on the analysis of single-modality EEG data in resting-state or auditory-evoked, which is relatively limited and cannot fully reflect the patient’s consciousness state. Multimodal EEG can reflect the function of different neural pathways in the brain from different perspectives and have complementary effects. Therefore, the combination of EEG features from different modalities can better characterize the residual consciousness of patients and further improve the accuracy of consciousness level assessment. However, the combined features can also generate feature redundancy, leading to overfitting of the classifier. Thus, optimization of the combined features is needed before building a classification model.

In order to address these issues, this study proposes a novel approach to assess the level of consciousness in DoC patients by combining spatiotemporal correlation entropy and neuromodulation intensity features in resting-state and auditory evoked EEG. Firstly, resting-state and auditory oddball paradigms were designed, and EEG data were recorded from 157 DoC patients. Secondly, nonlinear features of the resting-state EEG, as well as MMN and N1 features of the auditory-evoked EEG, were extracted and analyzed. The feature space composed of these features was then optimized using the multi-form feature selection algorithm. Finally, a support vector machine classifier was used to construct the consciousness evaluation model, which achieved relatively accurate grouping of DOC patients.

## 2 Materials and methods

### 2.1 Experimental design

#### 2.1.1 Experimental paradigm design

To obtain neural activity features of patients in the resting and auditory stimulation states, experimental paradigms were designed in this study:

##### 2.1.1.1 Resting-state

The EEG data was recorded during a 10-min period of the patient’s eyes-closed resting-state.

The subjects of this study are patients with cognitive impairment, whose active cognitive ability is severely impaired or even lost. The N1 and MMN components induced by the oddball paradigm can be generated only by passive stimulation. Compared with sensory-gated fMRI paradigms (Cecconi et al., 2024) that rely on automatic task design, More in line with this study.

##### 2.1.1.2 Oddball

**Figure 1:**
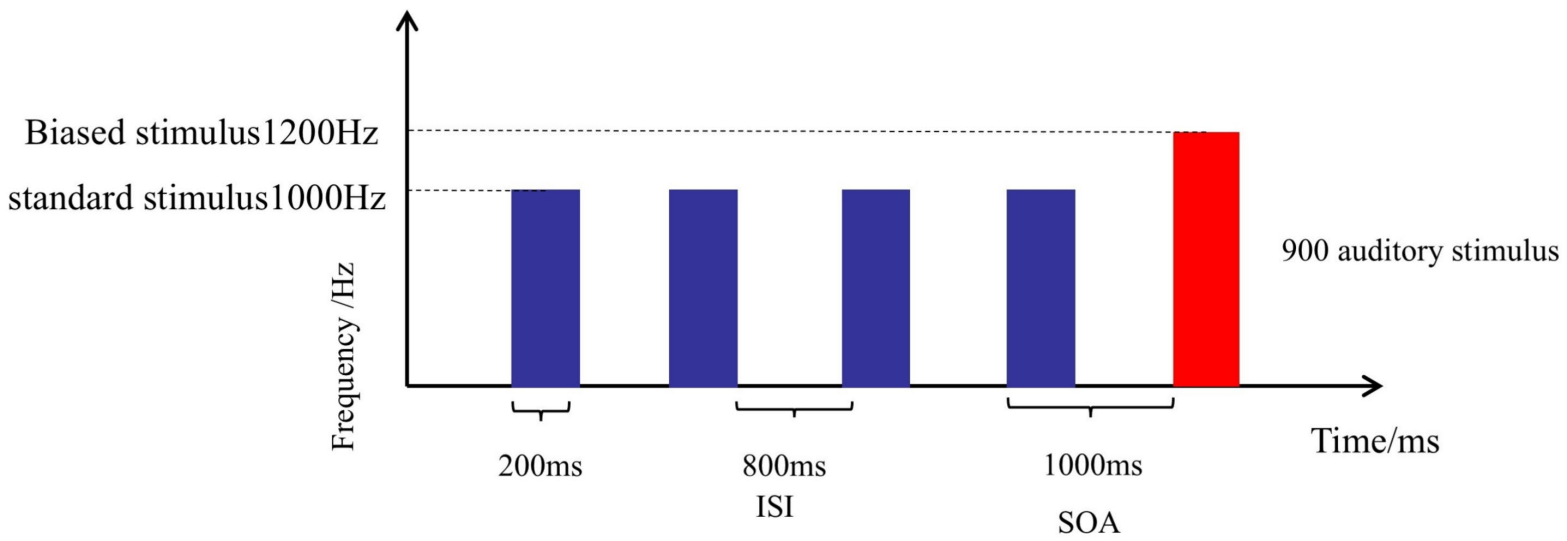
Oddball stimulus paradigm.

A pure tone at 1000 Hz was used as the standard stimulus (STD), and a pure tone at 1200 Hz was used as the deviant stimulus (DEV). The duration of all auditory stimuli was 200 ms, with a stimulus onset asynchrony (SOA) of 1,000 ms. The experimental paradigm consists of a total of 900 auditory stimuli presented in pseudorandom order, including 800 STD and 100 DEV, with at least 3 STD presented between two DEV stimuli. Programming control of the sound sequence was implemented using E-Prime 3.0 software. The duration of this experiment was approximately 20 min (Figure 1).

#### 2.1.2 Participants

We recruited 244 patients with disorders of consciousness for our study, but during the data collection process, 18 patients with MCS and 69 patients with UWS were excluded due to significant skull defects that led to excessive noise in the recorded EEG signals. Ultimately, we obtained EEG data from 157 patients, including 61 with MCS and 96 with UWS. Please refer to Table 1 for details about the patients. There were no significant differences in age or gender between the MCS and UWS groups. Prior to EEG recording, all patients underwent behavioral assessment using the CRS-R, which was evaluated multiple times by an experienced clinician.

**Table 1:** Information of DOC patients.

Group	*N*	Age	Gender	Etiology	Temporal criteria	CRS-R
DOC	157	56( ± 15)	108 M/49F	40 T/100H/17AN	8A/113P/36C	6.14( ± 3.51)
MCS	61	59( ± 14)	41 M/20F	18 T/35H/8AN	6A/43P/12C	9.5( ± 3.04)
UWS	96	56( ± 14)	67 M/29F	22 T/65H/9AN	2A/70P/24C	4.04( ± 1.69)

In Table 1, ‘A’ stands for acute (DOC duration less than 28), ‘AN’ stands for ANOXIC, ‘C’ stands for chronic (more than 3 months without trauma, more than 12 months with trauma), ‘F’ stands female, ‘M’ stands male, ‘P’ stands (3 more than 28 days), ‘T’ stands trauma, ‘H’ stands hemorrhage, ‘ ± ’ means standard deviation.

The diagnosis of consciousness relied on clinical consensus and CRS-R evaluation. Registered patients were hospitalized in the Trauma Surgery Center of a high-tech district hospital. Inclusion criteria were: (1) Diagnosis of UWS or MCS based on CRS-R behavioral evaluation, (2) Age over 16 years old, (3) No use of neuromuscular blockers or sedatives within 24 h after enrollment, (4) Presence of eye-opening (indicating normal sleep–wake cycles), and (5) Stable clinical condition.

This experimental design was in accordance with the Helsinki Declaration and had been approved by the Ethics Committee of Zhengzhou University Affiliated Central Hospital. Prior to the start of the experiment, patients’ family members were informed of the experimental tasks, and written informed consent was obtained from them.

#### 2.1.3 EEG data acquisition

The EEG data was acquired using a Nicolet V32 32-channel EEG recorder with electrode placement following the international 10/20 system. The channels included FP1, FP2, F3, F4, C3, C4, P3, P4, O1, O2, F7, F8, T3, T4, T5, T6, FZ, CZ, PZ, FC5, FC1, FC2, FC6, CP5, CP1, CP2, CP6, FPZ, POZ, OZ, A1, and A2. The EEG signal was sampled at a frequency of 1,000 Hz. The impedance of all electrodes was maintained below 5 KΩ. During the signal acquisition process, the indoor environment was kept quiet and free of other electromagnetic interference. The reference electrodes used for EEG data acquisition were A1 and A2.

### 2.2 EEG signal pre-processing

**Figure 2:**
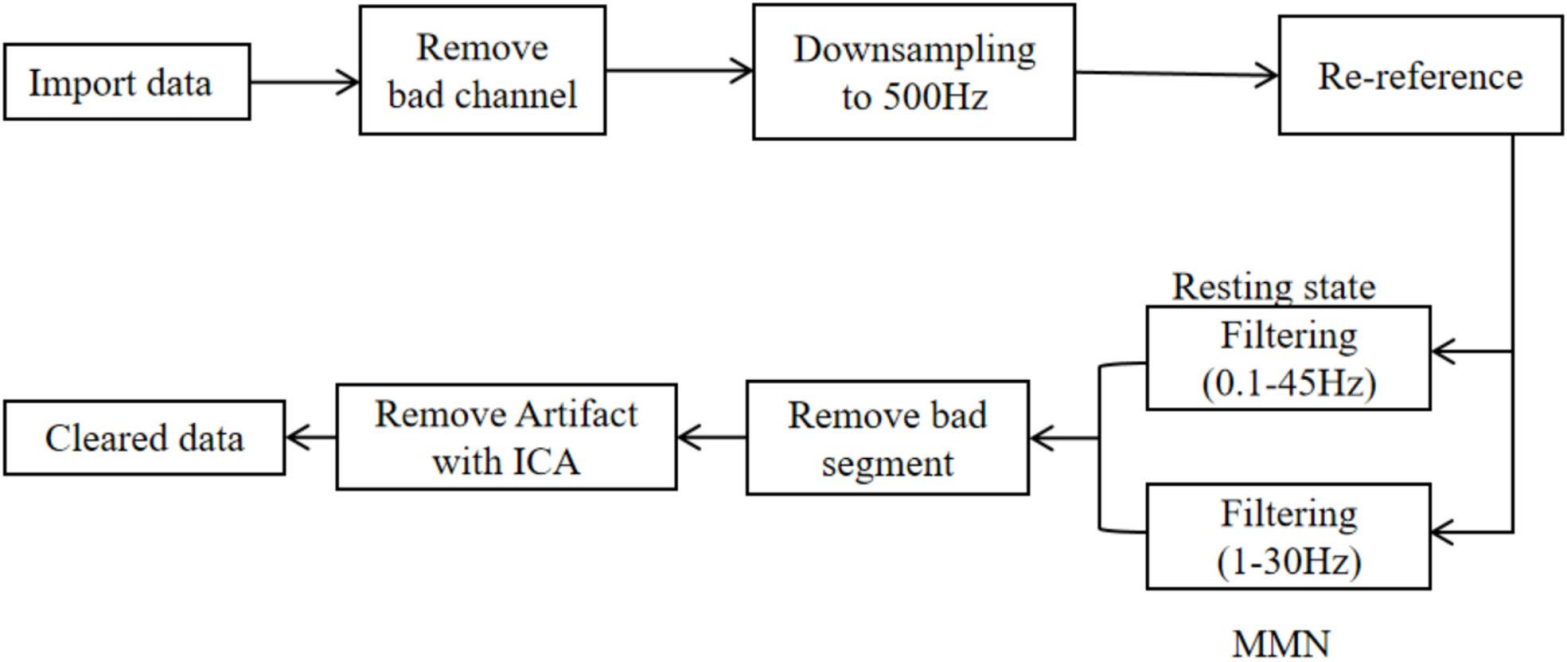
Preprocessing frame diagram for resting-state and auditory-evoked EEG signals.

The EEG preprocessing pipeline is illustrated in Figure 2 and consists of the following main steps: (1) importing the raw data and removing bad channels, (2) down sampling the data from 1,000 Hz to 500 Hz and re-referencing it to common average reference (Hu et al., 2017), (3) applying Butterworth bandpass filtering with cutoff frequencies of 0.1–45 Hz for the resting-state EEG and 1–30 Hz for the auditory-evoked EEG, (4) manually removing bad segments, and (5) performing independent component analysis(ICA) to remove artifacts and obtain cleaned data.

### 2.3 EEG data analysis and feature extraction

#### 2.3.1 NDF analysis

EEG exhibits strong nonlinear and dynamic characteristics, which can reflect the complexity of the patient’s brain state. We divided the EEG data into the aforementioned five frequency bands and calculated Spatiotemporal correlation entropy and Neuromodulations intensity for each patient.

##### 2.3.1.1 Spatiotemporal correlation entropy

The spatiotemporal correlation entropy based on the definition of correlation entropy and the two-component hypothesis (Freeman and Quiroga, 2012) can be used to quantify brain region synchronization. The dynamic changes of brain synchronization can effectively reflect the level of consciousness (Bharath et al., 2017). The two-component hypothesis decomposes EEG signals into the neuromodulation activities and background activities. The probability density function of EEG signals during the neuromodulation activity is significantly deviated from a Gaussian distribution, while the probability density function during other phases complies with a Gaussian distribution. Correlation entropy can be used to map the statistical difference in the distributions of the two components since it makes use of the probability density functions to measure the similarity between two random variables. It introduces nonlinearity to provide the even order moment of the joint probability density function of the two random variables. Based on the two-component hypothesis. As a first step, we decompose the multi-channel EEG data X into two matrix components using Equation 1.


X=L+S (1)

In this equation, L is a low-rank matrix related to background activity stability, S is Sparse matrix related to transient neuromodulation. L and S have different statistical properties, so L is not affected by S during the same time period. In order to estimate neuromodulations activity, We divide X into N consecutive time segments, $X=[X_{1},X_{2},\ldots ,X_{N}]$ , where each $X_{n}$ represents a multi-channel signal lasting T seconds. The number of segments N is obtained by dividing the total duration of X by T , we will set the segment length T to 2 s. The signal of each channel in $X_{n}$ is expressed as $X_{n,j}$ , where j represents the channel number. Entropy $C_{n,j}$ of each channel in the time domain. The calculation formula is Equation 2:


$$C_{n,j}=\frac{1}{t*\mathrm{fs}}\sum_{i=1}^{N}\sum_{k=1}^{t*\mathrm{fs}}\exp(-\frac{1}{2\sigma_{1}^{2}}{\left(X_{n,j,k}-X_{i,j,k}\right)}^{2})$$ (2)

In this formulation, $X_{i,j,k}$ denotes the k -th time point of the j -th channel in the i -th signal segment, $f_{s}$ represents the sampling frequency, and $\sigma_{1}$ denotes a Gaussian kernel with kernel width $\sigma_{1}$ . The distributional similarity between the n−th segment $X_{n,j}$ and all other segments $X_{i,j,}:(i=1\cdots n)$ on the same channel j is computed and accumulated, yielding $C_{n,j}$ , which quantifies the similarity between the n−th segment and the remaining segments of channel j .

Stable and temporally dense background activity is expected to result in larger values of $C_{n,j}$ , whereas neuromodulator-related activity, being sparse and non-stationary, produces smaller values of $C_{n,j}$ . This approach thereby incorporates temporal characteristics into the estimation of distributional similarity. Consequently, a matrix $C_{N}$ can be constructed, which comprises the time-entropy sequence of all channels such as Equation 3.


$$C_{N}=(\begin{array}{ccc} C_{1,1} & \cdots & C_{N,1}\\ \vdots & \ddots & \vdots \\ C_{1,J} & \cdots & C_{N,J} \end{array})$$ (3)

Then, consider the spatial correlation coefficient, that is, the similarity of the temporal correlation coefficient sequences between different channels. The calculation method is as shown in Equation 4:


$$Z_{j}=\sum_{k=1}^{J}\sum_{n=1}^{N}\exp(-\frac{1}{2\sigma_{1}^{2}}{\left(C_{n,j}-C_{n,k}\right)}^{2})$$ (4)

In this equation, $Z_{j}$ represents the sum of the time correlation coefficient similarities between the *j*-th channel and all other channels, which is the Spatiotemporal correlation coefficient of a channel. Therefore $Z=[Z_{1},Z_{2},\cdots Z_{J}]$ represents the measurement of the spatiotemporal correlation coefficient of the entire brain, where *J* is equal to the total number of channels.

Neuromodulation Intensity: Based on the definition of correlation entropy and the two-component hypothesis, this study estimates the proportion of neuromodulatory activity on each channel as a measure of neuromodulation intensity for assessing the level of consciousness. Patients with higher levels of consciousness are hypothesized to exhibit a greater likelihood of neuromodulatory activity.

Following the decomposition algorithm derived from the temporal correlation coefficient C and neuromodulatory activity on a single channel (Freeman and Quiroga, 2012), neuromodulatory activity (S) and background activity (L) are separated through a percentile-based analysis combined with a skewness test of Gaussianity. Specifically, the background activity $L_{j}$ on channel j is defined as the signal segment with the minimum skewness, while the neuromodulatory activity $S_{j}$ corresponds to the remaining signal segments that deviate from Gaussianity.

The proportion of $S_{j}$ among all signal segments on channel j is denoted by $P_{j}$ , representing the probability of neuromodulatory activity on that channel. By aggregating the channel-wise probabilities into a vector $P=[P_{1}P_{2}\cdots P_{J}]$ , the overall neuromodulation intensity can be obtained, which serves as an indicator of the global level of consciousness. The calculation method is as shown in Equations 5–10:


$$L(\rho )=[X_{n,j},n\in \arg(C_{\left(:,j\right)}>\text{percentile}(C_{\left(:,j\right)},\rho ))]$$ (5)

$$\rho^{*}=\text{argmin}(|\text{skewness}(L(\rho ))|)$$ (6)

$$\text{Skewness}(L(\rho ))=\frac{1}{N\cdot \sigma^{3}}\sum_{i=1}^{N}\;{\left(x_{i}-\mu \right)}^{3}$$ (7)

$$L_{j}=L(\rho^{*})$$ (8)

$$S_{j}=[X_{n,j},n\in \arg(C_{\left(:,j\right)}<\text{percentile}(C_{\left(:,j\right)},\rho^{*}))]$$ (9)

$$P_{j}=|S_{j}|/(|S_{j}|+|L_{j}|)$$ (10)

In this article, we will the search range of ρ is set to 10%~90% , the step size is set to 5%.

#### 2.3.2 ERP analysis

First, the preprocessed EEG data during the auditory stimulation state were segmented into 500 ms epochs, including 100 ms pre-stimulus and 400 ms post-stimulus data. Then, baseline correction was performed on all trials, and trials with amplitudes exceeding 75 μV after correction were rejected. Finally, the ERP waveform was obtained by averaging each segment of data, and the MMN component was calculated by subtracting the ERP waveform elicited by the standard stimulus from that elicited by the deviant stimulus. In order to obtain more stable ERP components, wavelet filtering was applied to the segmented data (Cong et al., 2015).

In the passive auditory ERPs experiment, the focus was mainly on the N1 and MMN components in the STD trials, and this study analyzed the differences in amplitude and latency of N1 and MMN components between the two groups of patients. The measurement method for N1/MMN component amplitude was the peak corresponding to the N1/MMN component, and the time point of the peak was the latency.

Influenced by the resting-state SC and NI theories, this article calculated SC (ESC) and NI (ENI) under the passive auditory ERP paradigm (The calculation method is the same as the resting state). In order to compare the changes in the spatiotemporal complexity of the brain after the patient received auditory stimulation, this article also calculated SC and NI in the full frequency range under resting-state EEG. According to the Oddball paradigm in this article, the duration of the standard stimulus and the deviation stimulus are both 1 s, so when calculating ESC and ENI, we set the segment length to 1, that is, only calculate SC and NI under auditory stimulation.

### 2.4 Classification based on MFFS and SVM

In this study, NDF, and ERP features were combined to form a feature space, but there were still feature redundancies in the feature space. Therefore, we combined the multi-form feature selection algorithm (MFFS) with support vector machine (SVM) classifiers to find the feature combination with the highest classification accuracy (Jiao et al., 2022). MFFS is an evolutionary algorithm that applies multiple evolutionary forms to more efficiently search for feature subsets. It balances convergence and diversity by combining different evolutionary forms, making it suitable for exploring complex interactions between features and selecting high-accuracy feature combinations.

To validate the performance of the classifier, a five-fold cross-validation was conducted with a population size of 200, consisting of two subpopulations of 100, and 100 iterations. In each round, one of the subsets is selected as the validation set, and the remaining four subsets are used as training sets. In MFFS, each feature was represented by a chromosome. There were 102 features in total, encoded as 0 or 1, and each chromosome was represented by a 102-bit binary number. First, the feature set was input into the established multi-form task, and then the population of MFFS was initialized. The classifier uses the SVM classifier with radial basis kernel and the penalty coefficient is set to 1.

**Figure 3:**
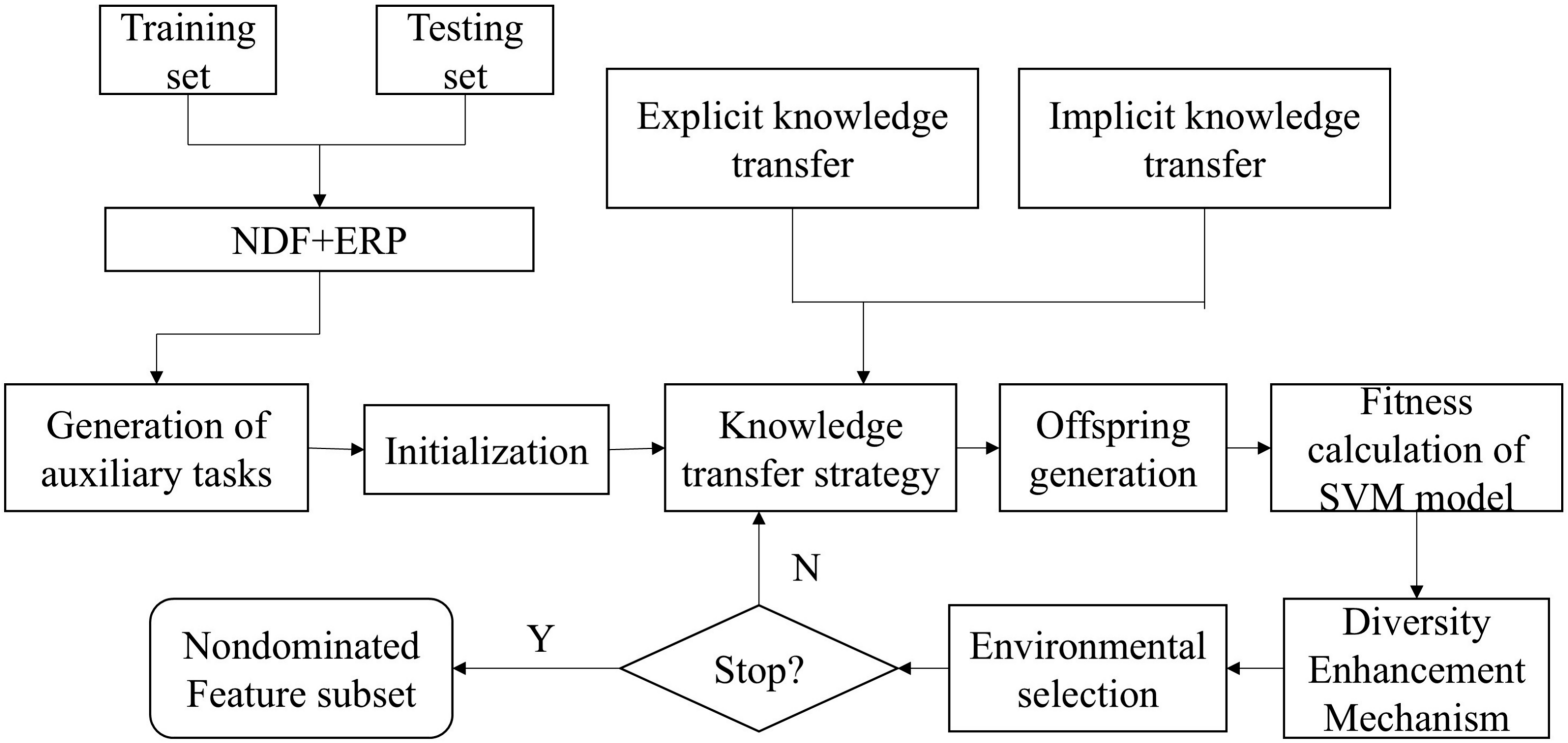
Feature selection based on MFFS and SVM framework.

Two knowledge transfer mechanisms guide the population evolution, which were applied to offspring generation (implicitly) and environmental selection (explicitly), respectively. Implicit knowledge transfer was achieved by passing effective feature combinations searched by one form of population to the other population through breeding operations. Explicit knowledge transfer was achieved by migrating the elite solutions of one form of population to guide the evolution of the other population. The newly generated offspring after knowledge transfer learning were input into the SVM to calculate the classification accuracy, and the classification error was used as the fitness value of the individual. Finally, the next generation was obtained through environmental selection based on the Chebyshev method. After iteration, the individual with the highest classification accuracy in the first Pareto front of the population was retained as the optimal solution. The specific process is shown in the Figure 3.

### 2.5 Statistical analysis

We conducted a two-sample t -test to analyze the differences in the proposed features between the two patient groups, with a significance level of 0.05. The false discovery rate (FDR) was used to correct for multiple comparisons across channels.

## 3 Results

### 3.1 Results of NDF parameters

Based on the current research, we set $\sigma_{1}$ to $\frac{\sigma^{*}}{1.5}$ , where $\sigma^{*}$ denotes the bandwidth estimated using Silverman’s rule (Silverman, 2018). According to the above theoretical principles of spatiotemporal entropy and neuromodulation intensity, we first calculate the spatiotemporal entropy and neuromodulation intensity values of each lead are calculated, respectively. After that the average value of all channels is calculated to summarize the spatial information.

**Figure 4:**
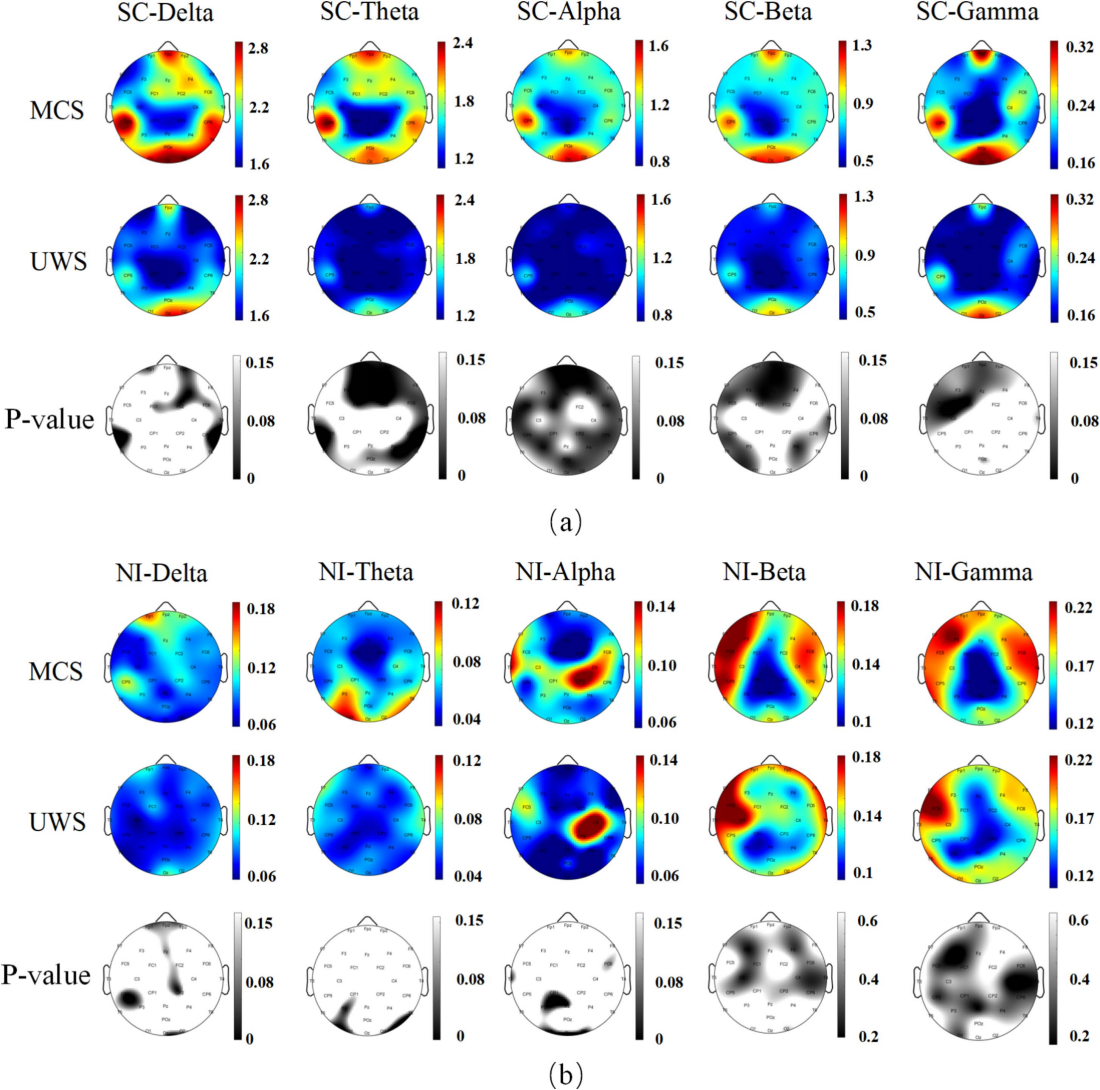
Brain topography of SC and NI calculation results in DOC patients. **(a)** Brain topography of the difference between SC and SC in five frequency bands of the whole brain in MCS and UWS; **(b)** Brain topography of the difference between NI and NI in five frequency bands of the whole brain in MCS and UWS.

Figure 4 presents the brain topography of patients in the UWS group and the MCS group. The maps are generated from EEG recordings and display two key parameters—Spatiotemporal Correlation Entropy (SC) and Neuromodulation Intensity (NI)—across five frequency bands: Delta (1–4 Hz), Theta (4–8 Hz), Alpha (8–13 Hz), Beta (13–30 Hz), and Gamma (30–45 Hz).

The visualization shows that both SC and NI values are higher in the MCS group compared to the UWS group across all five frequency bands. From the color intensity of the topographic maps, it is clear that SC provides a stronger group distinction than NI. In addition, SC values in the Gamma band are noticeably lower than those observed in the other frequency bands.

In order to quantitatively evaluate the statistical differences between the UWS group and the MCS group, we conducted an independent sample *t-*test for all channels.

As shown in the third row of Figure 4, the SC values differ significantly (*p* < 0.05) between the UWS and MCS groups across all five frequency bands. These differences mainly appear in the brain regions highlighted by the darker areas on the topographic maps. Among the five bands, the Theta, Alpha, and Beta bands show a larger number of channels with significant group differences compared to the Delta and Gamma bands.

In contrast, for NI values, significant differences (*p* < 0.05) between the two groups are only observed in the low-frequency bands (Delta, Theta, and Alpha). In the higher-frequency bands (Beta and Gamma), no channels show significant group differences.

**Figure 5:**
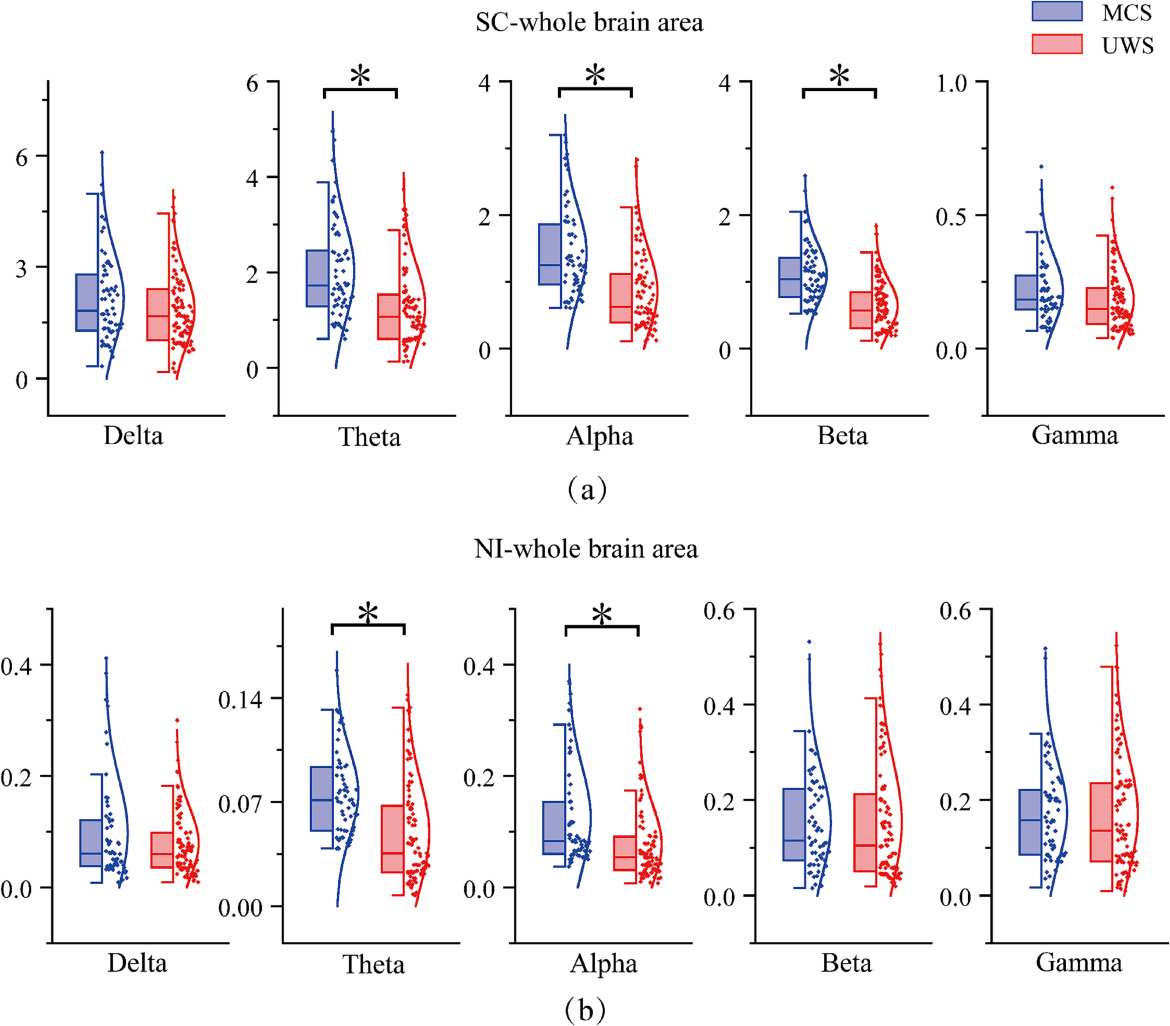
Results chart of significant difference between SC and NI whole brain regions in DOC patients. Statistical significance was indicated by * (*p*<0.05, FDR corrected), **(a)** SC results of 5 frequency bands in the whole brain. **(b)** NI results of 5 frequency bands in the whole brain.

We then averaged the measurements across all EEG channels to obtain whole-brain values, providing a preliminary quantitative way to distinguish between groups with different levels of consciousness. As shown in Figure 5, statistical analysis using independent samples t-tests revealed that both SC and NI values in the Theta and Alpha frequency bands were significantly different (*p* < 0.05) between the two groups of patients. In addition, SC values also showed significant differences in the Beta band (*p* < 0.05). A detailed summary of the whole-brain SC and NI results for both groups is provided in Tables 2, 3.

**Table 2:** Summary of statistical test results of SC whole brain region.

Parameter	Frequency band	Mean difference	Standard error difference	H	*p*-value
Whole brain region	Delta	0.3124	0.1945	0	0.110
	Theta	0.7183	0.1775	1	<0.001
	Alpha	0.5886	0.1149	1	<0.001
	Beta	0.4746	0.0846	1	<0.001
	Gamma	0.0385	0.0212	0	0.072

**Table 3:** Summary of statistical test results of NI whole brain region.

Parameter	Frequency band	Mean difference	Standard error difference	H	*p*-value
Whole brain region	Delta	0.0099	0.0148	0	0.504
	Theta	0.0112	0.0013	1	0.013
	Alpha	0.0453	0.0136	1	0.001
	Beta	−0.0021	0.0198	0	0.917
	Gamma	0.0046	0.0186	0	0.804

**Figure 6:**
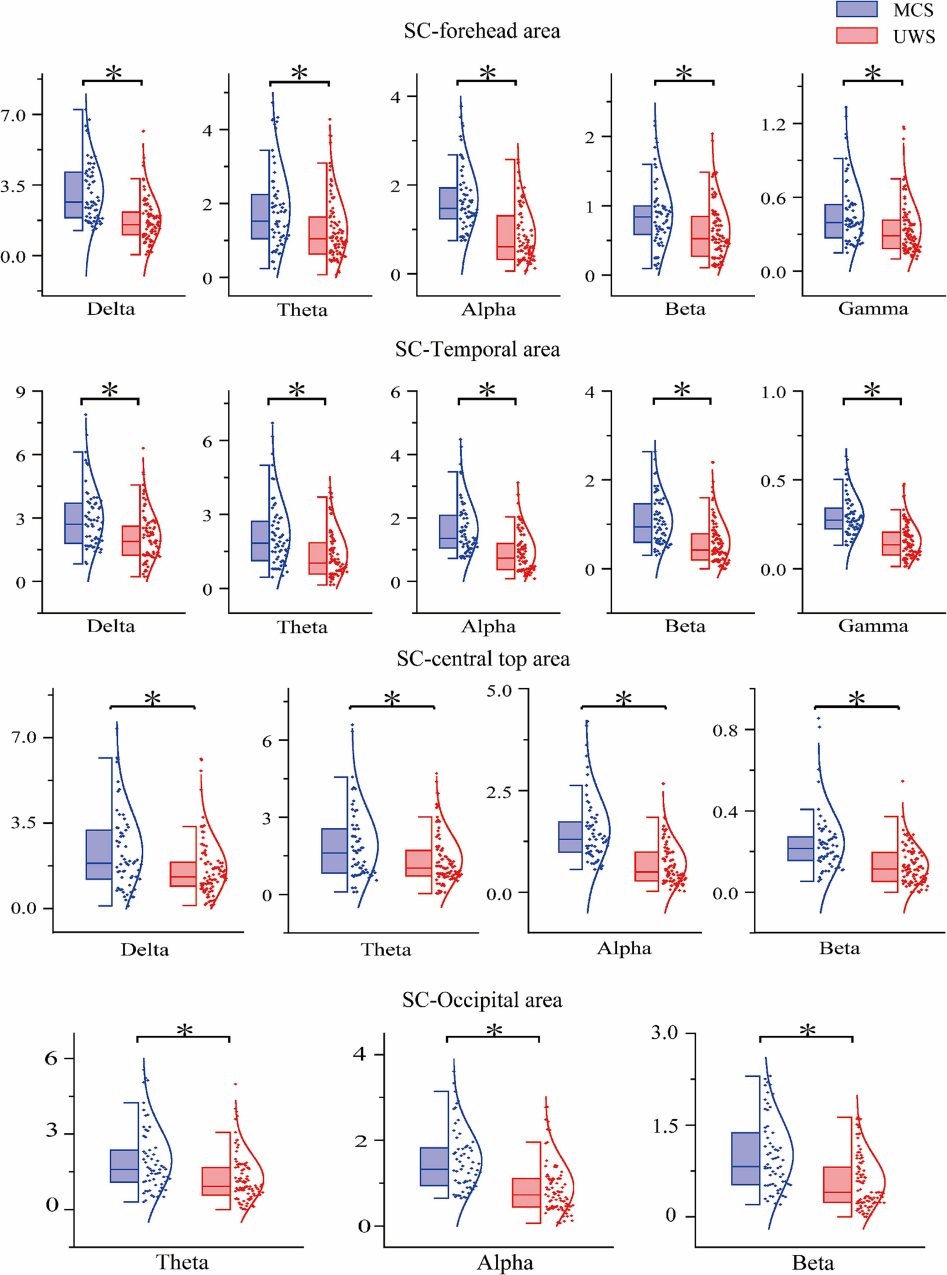
Results chart of significant differences in different brain regions of SC in DOC patients.

To further explore how SC and NI values vary across different brain regions and EEG frequency bands, we performed statistical analyses on data from four regions: the frontal, central-parietal, temporal, and occipital areas (Figure 6).

For SC values, significant differences (*p* < 0.05) between the UWS and MCS groups were found in the Theta, Alpha, and Beta bands across all four brain regions. Although no significant group differences were observed for Delta and Gamma bands at the whole-brain level, regional analysis revealed that Delta-band SC values were significantly different in the frontal, central-parietal, and temporal regions (but not in the occipital region). In addition, Alpha-band SC values also showed strong discriminatory power in the frontal and temporal regions.

**Figure 7:**
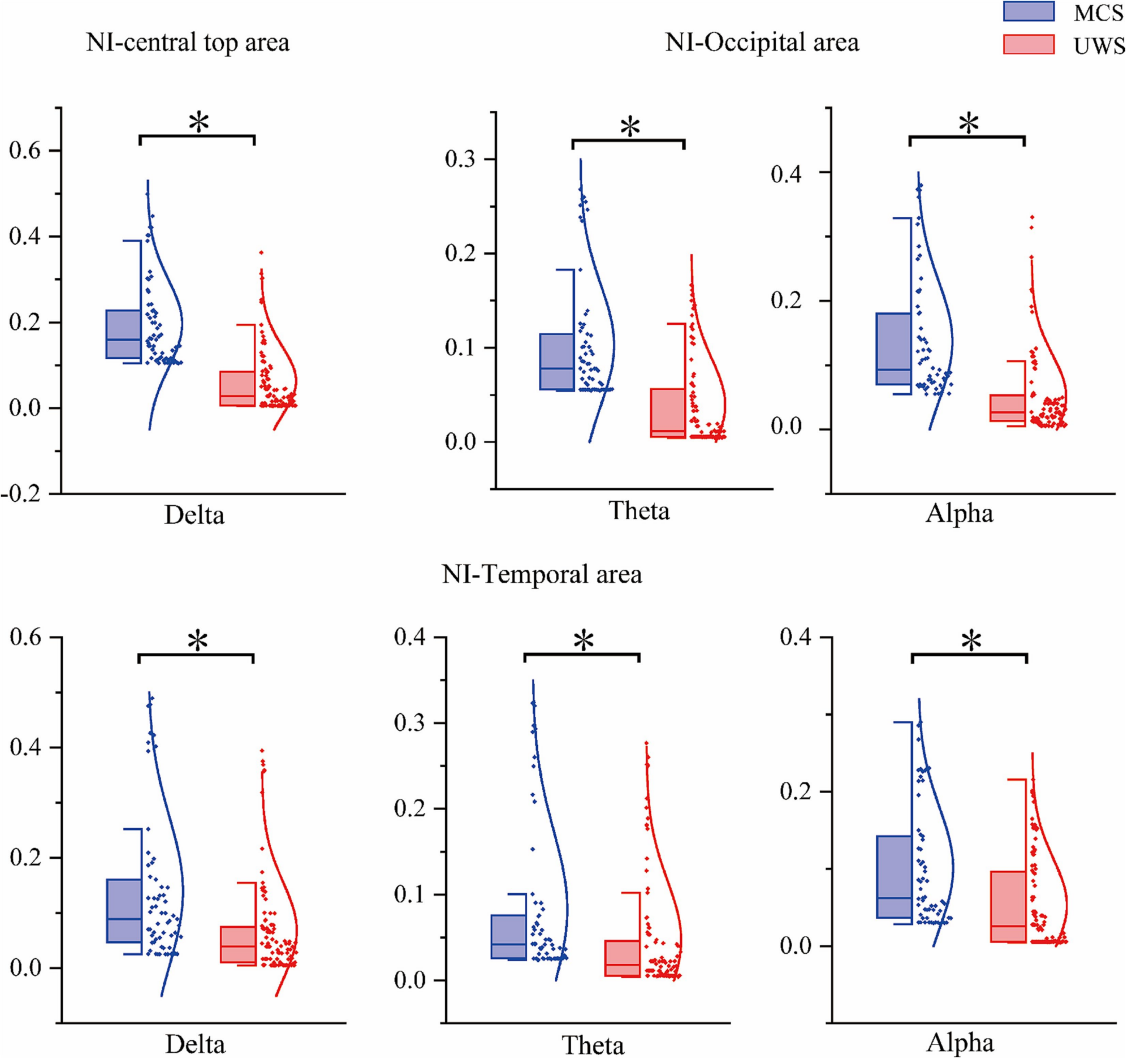
Results chart of significant differences in different brain regions of NI in DOC patients.

As shown in Figure 7, the visualization indicates that, compared with SC, the ability of NI to distinguish between the two patient groups is noticeably weaker. Significant differences (*p* < 0.05) were only found in specific regions: in the Delta band, differences appeared in the temporal and central-parietal areas; in the Theta and Alpha bands, differences were observed in the temporal and occipital areas. For other frequency bands and brain regions, NI showed no significant group differences. Detailed statistical results for SC and NI across different brain regions are summarized in Tables 4, 5.

**Table 4:** Summary of statistical test results in different brain regions of SC.

Parameter	Frequency band	Mean difference	Standard error difference	H	*p*-value
Forehead area	Delta	1.5019	0.2376	1	<0.001
	Theta	0.6233	0.1811	1	<0.001
	Alpha	0.8662	0.1187	1	<0.001
	Beta	0.2532	0.0884	1	0.005
	Gamma	0.1395	0.0437	1	0.002
Central top area	Delta	0.7581	0.2399	1	0.002
	Theta	0.5438	0.1718	1	0.005
	Alpha	0.8119	0.1237	1	<0.001
	Gamma	0.1155	0.0221	1	<0.001
Temporal area	Delta	0.8812	0.2242	1	<0.001
	Theta	0.7597	0.2024	1	<0.001
	Alpha	0.7415	0.1269	1	<0.001
	Beta	0.5327	0.1004	1	<0.001
	Gamma	0.1525	0.0214	1	<0.001
Occipital area	Theta	0.7525	0.2136	1	<0.001
	Alpha	0.6281	0.1270	1	<0.001
	Beta	0.4178	0.0958	1	<0.001

**Table 5:** Summary of statistical test results of different brain regions in NI.

Parameter	Frequency band	Mean difference	Standard error difference	H	*p*-value
Central top area	Delta	0.1443	0.0205	1	<0.001
Temporal area	Delta	0.0822	0.0239	1	<0.001
	Theta	0.0694	0.0247	1	0.006
	Alpha	0.0911	0.0293	1	0.002
Occipital area	Theta	0.1069	0.0235	1	<0.001
	Alpha	0.0936	0.0148	1	<0.001

### 3.2 Results of MMN and N1

**Figure 8:**
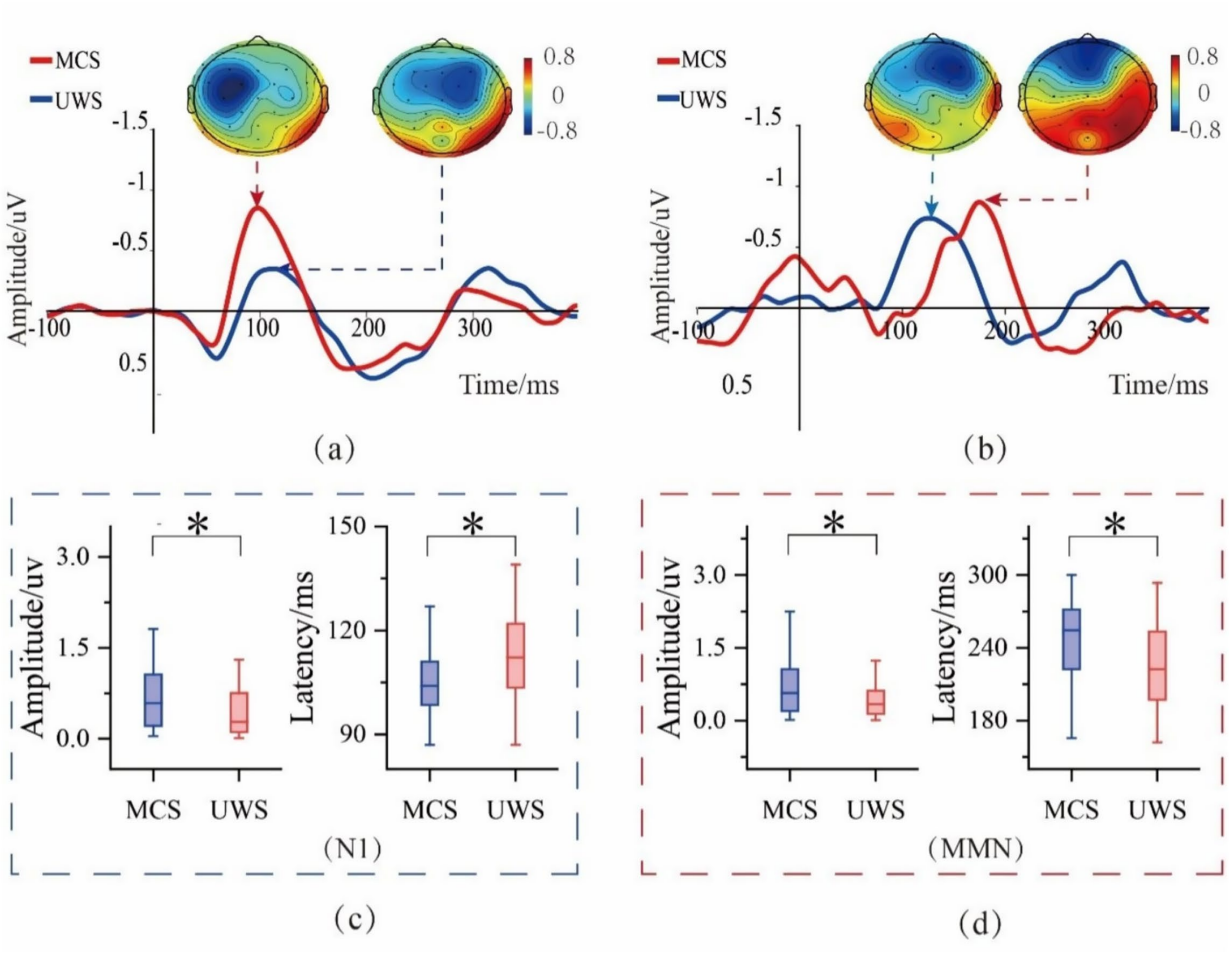
Analysis results of N1 and MMN components. **(a)** Grand average waveforms and corresponding topographical maps of the N1 component at the C3 electrode. **(b)** Grand average waveforms and corresponding topographical maps of the MMN component at the Fz electrode. **(c)** Boxplot of the mean amplitude and latency of the N1 component at channels with significant differences. **(d)** Boxplot of the mean amplitude and latency of the MMN component at channels with significant differences (* indicates *p*<0.05, FDR corrected).

Figure 8 illustrates the results for the N1 and MMN components. Figure 8a shows the grand-averaged waveform and brain topography of the N1 component at the C3 electrode, while Figure 8b presents the corresponding results for the MMN at the Fz electrode. Figures 8c,d display the outcomes of the two-sample *t*-tests.

Under standard stimulus (STD) conditions, the absolute amplitude of the N1 component was significantly larger in MCS patients compared with UWS patients (*p* < 0.05, FDR corrected), with differences observed at the C3, Cz, and FC2 electrodes. In addition, the N1 latency was significantly longer in UWS patients than in MCS patients (*p* < 0.05, FDR corrected), and this effect was evident at the C3, C4, Cz, and FC5 electrodes.

Under deviant stimulus (DEV) conditions, the mismatch negativity (MMN) component showed a similar pattern: MCS patients exhibited significantly higher absolute amplitudes than UWS patients (*p* < 0.05, FDR corrected), with differences found at the C3, F3, FC5, FC1, and Fz electrodes. However, unlike the N1 component, the MMN latency was longer in MCS patients compared with UWS patients (*p* < 0.05, FDR corrected), with significant differences at the C3, Cz, FC2, and FC1 electrodes.

### 3.3 SC and NI result of auditory-evoked EEG

**Figure 9:**
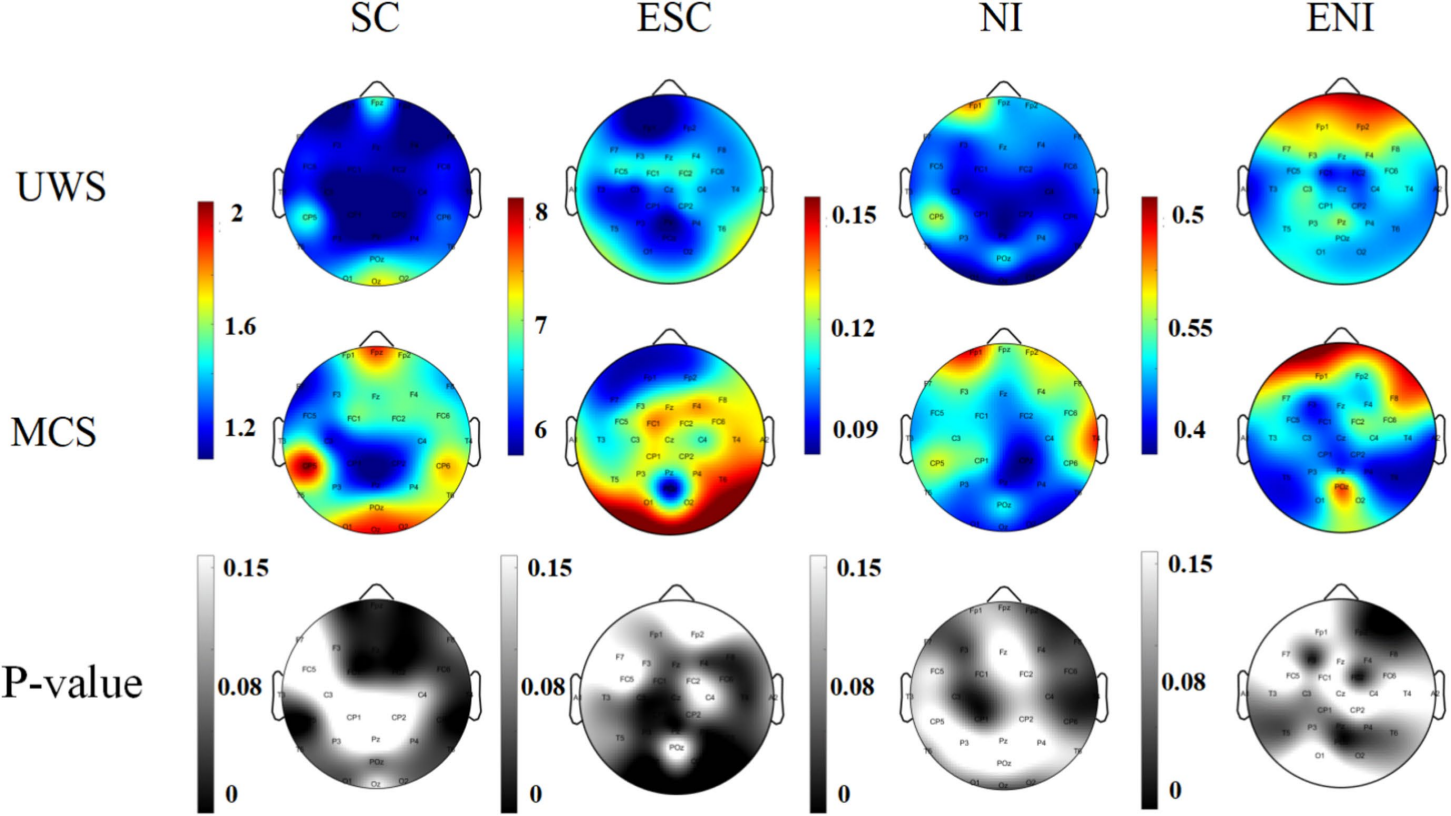
Analysis of spatiotemporal complexity, neuromodulation intensity and difference brain topography in resting state and ERP patients with DOC.

To assess the validity of the calculated ESC and ENI measures under auditory stimulation, we first plotted the corresponding brain topographies to show their spatial distribution at the group level. As shown in the first two rows of Figure 9, both ESC and ENI exhibit the same overall trend as SC and NI across the full frequency band, with higher values observed in MCS patients compared to UWS patients.

We then performed independent-samples t-tests on all channels to quantitatively evaluate group differences. As shown in the third row of Figure 9, significant differences were found after auditory stimulation, and the distribution of these differences was distinct from those observed in the resting state. In the resting state, SC and NI differences were mainly concentrated in the frontal and central-parietal regions. In contrast, under auditory stimulation, ESC differences were most pronounced in the central-parietal and occipital regions. ENI showed significant differences not only in the central-parietal region but also in occipital channels that were able to distinguish between the two groups.

Finally, to summarize the spatial information and provide a preliminary quantitative index of group differentiation, we calculated average measurements across all channels.

**Figure 10:**
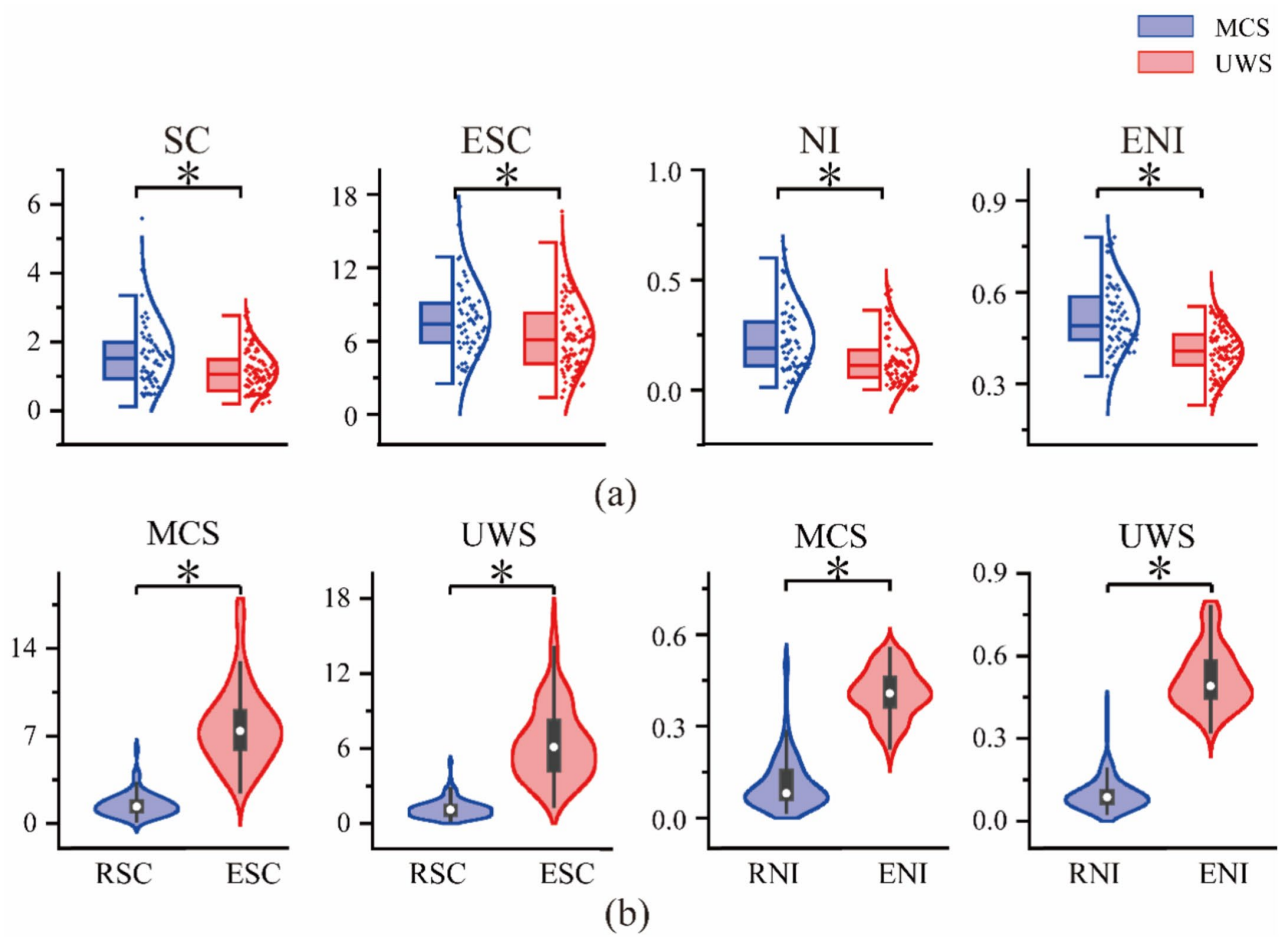
Full-band analysis of ESC and ENI in DOC patients, **(a)** Comparison of the spatiotemporal correlation entropy and neuromodulation intensity box plots between MCS and UWS in resting and task states; **(b)** Violin plots of spatiotemporal correlation entropy and neuromodulation strength of MCS and UWS in the resting state and task state comparisons.

As shown in Figure 10a, statistical analysis of the average measurements revealed significant differences between UWS and MCS patients for both ESC and ENI, as well as for SC and NI across the full frequency band (*p* < 0.05). We also compared these measures with their resting-state counterparts, resting-state spatiotemporal entropy (RSC) and resting-state neuromodulation intensity (RNI). As shown in Figure 10b, ESC and ENI values were significantly higher than RSC and RNI. Independent-samples t-tests confirmed that these differences were statistically significant (*p* < 0.01), indicating that the brain’s spatiotemporal complexity increases markedly under auditory stimulation.

Consistent with the trends seen in the brain topographies, the mean ESC and ENI values increased with higher levels of consciousness. These results clearly demonstrate that the proposed method can effectively distinguish between patient groups with different levels of consciousness, highlighting significant differences between MCS and UWS.

As shown in Figure 10a, statistical analysis of the average measurement values revealed significant differences between UWS and MCS patients for both ESC and ENI, as well as for SC and NI across the full frequency band (*p* < 0.05). We further compared the resting-state measures—resting-state spatiotemporal entropy (RSC) and resting-state neuromodulation intensity (RNI)—with the ESC and ENI values. As illustrated in Figure 10b, ESC and ENI were significantly higher than RSC and RNI. Independent-samples t-tests confirmed that these differences were statistically significant (*p* < 0.01), indicating a marked increase in the spatiotemporal complexity of the brain under auditory stimulation.

Consistent with the monotonic trends observed in the brain topographies, the mean values of ESC and ENI increased with higher levels of consciousness. These findings strongly support the effectiveness of the proposed method in distinguishing patient groups with different levels of consciousness and highlight clear differences between MCS and UWS.

### 3.4 Results of diagnostic model

We attempted to solve the classification problem of patients with different levels of consciousness disorders by using NDF, and ERP components. We selected the optimal classification features and combined the feature parameters to improve the classification accuracy of the MCS and UWS groups as much as possible. The feature set used in this study is shown in Table 6.

**Table 6:** Classification results based on EEG feature optimization.

Feature combination	Feature selection	Average accuracy
Resting-state EEG characteristics	SC-Delta, SC-Theta, SC-Alpha, SC-Beta, SC-Gamma, NI-Delta, NI-Theta, NI-Alpha, NI-Beta, NI-Gamma	86.6%
ERP characteristics	N1-Amplitude, N1-Latency, MMN-Amplitude, MMN-Latency, ESC, ENI	82.2%
Resting-state + ERP characteristics	SC-Delta, SC-Theta, SC-Alpha, SC-Beta, SC-Gamma, NI-Delta, NI-Theta, NI-Alpha, NI-Beta, NI-Gamma, N1-Amplitude, N1-Latency, MMN-Amplitude, MMN-Latency, ESC, ENI	89.8%
MFFS-SVM	SC-Theta, SC-Alpha, NI-Alpha, N1-Amplitude, MMN-Amplitude, MMN-Latency, ESC, ENI	92.4%

In order to compare the classification performance of multi-modal EEG features, we first extract and select separate resting-state and ERP features for classification. The average classification accuracies are 86.6 and 82.2%, respectively. Resting-state EEG features were found to have high classification accuracy. Next, this article combines the above resting-state and ERP characteristics for classification, and the classification accuracy reaches 89.8%. Next, this article combines the above resting-state and ERP features for classification, and the classification accuracy reaches 89.8%, which is higher than the accuracy of single-type feature classification. However, simple feature combination may lead to feature redundancy, requiring further feature optimization of the combined features.

**Figure 11:**
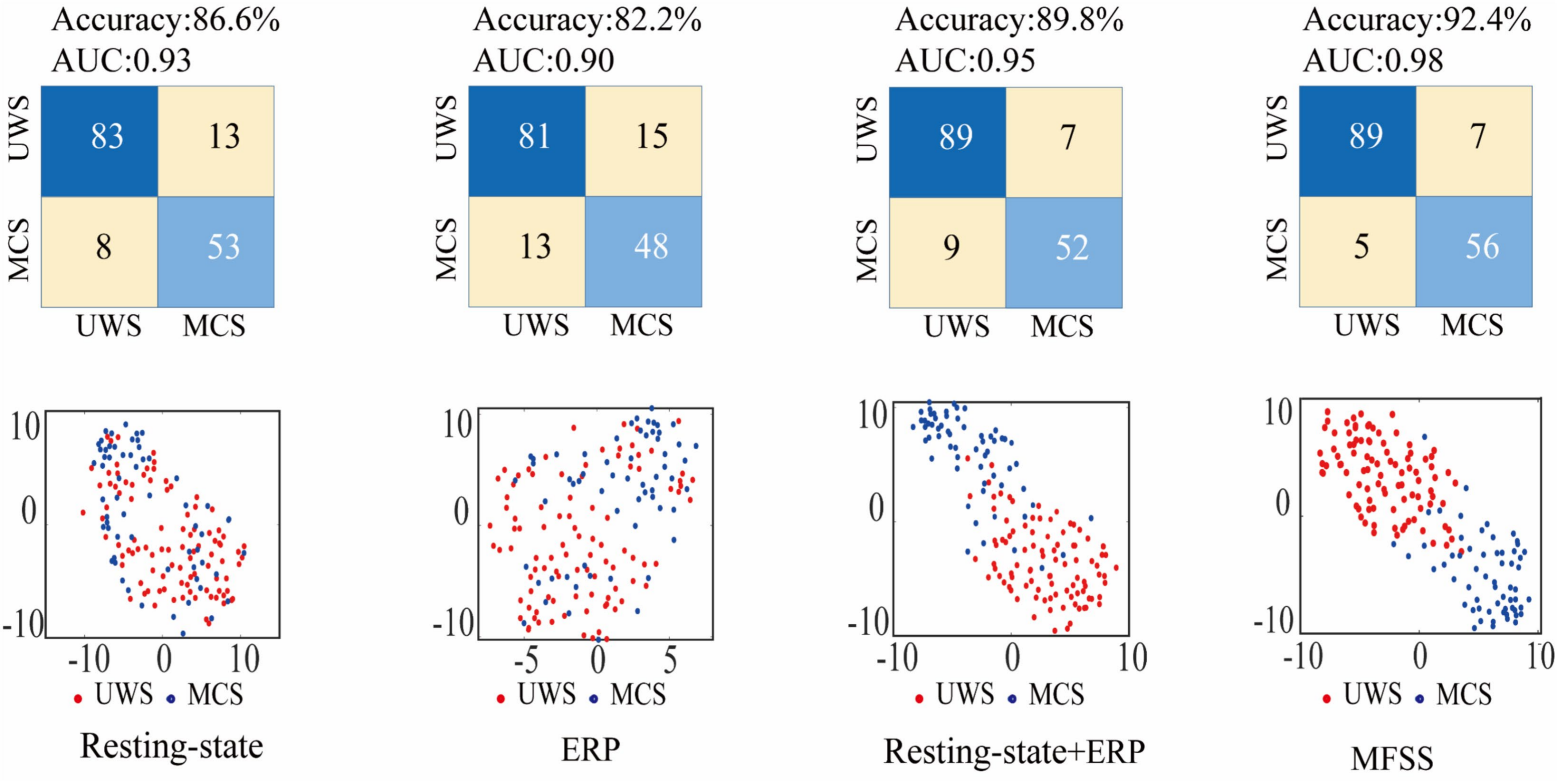
Confusion matrix and visualization diagram of classification results of different feature sets. Among them, the horizontal and vertical coordinates of different feature classification visualizations are feature dimensions.

Finally, we apply MFFS to optimize the extracted features, resulting in an optimal feature set containing eight features — three resting-state EEG features (SC-Theta, SC-Alpha, and NI-Alpha) and five ERP features. Features (N1-Amplitude, MMN-Amplitude, MMN-Latency, ESC and ENI), the classification accuracy reaches 92.4%. To visually compare the performance of the optimized combined features and the original features, we used t -SNE ( t -Distributed Stochastic Neighbor Embedding) to visualize the feature data (Van der Maaten and Hinton, 2008). The classification results and confusion matrix of different feature selections and their corresponding visualization are shown in Figure 11. The results show that the discriminative ability of the optimized combined features is significantly higher than that of the original features, indicating that the combination of multi-dimensional EEG features can effectively improve the diagnosis of patients with different degrees of consciousness disorders.

To further evaluate the effectiveness of the feature set that combines resting-state and auditory-evoked EEG features and is optimized using MFFS, the classification performance metrics calculated in this study are presented in Table 7. The results indicate that the MFFS-optimized feature set outperforms all other sets across all indicators, followed by the combined resting-state + ERP features. Resting-state EEG features alone perform slightly worse, while ERP features alone show the lowest performance.

**Table 7:** Performance metrics for different feature sets.

Feature combination	Specificity	Recall	Accuracy	F1 score	AUC
Resting-state	0.912	0.803	0.866	0.835	0.95
ERP	0.862	0.762	0.822	0.774	0.90
Resting-state + ERP	0.908	0.881	0.898	0.866	0.95
MFFS	0.947	0.889	0.924	0.903	0.98

**Figure 12:**
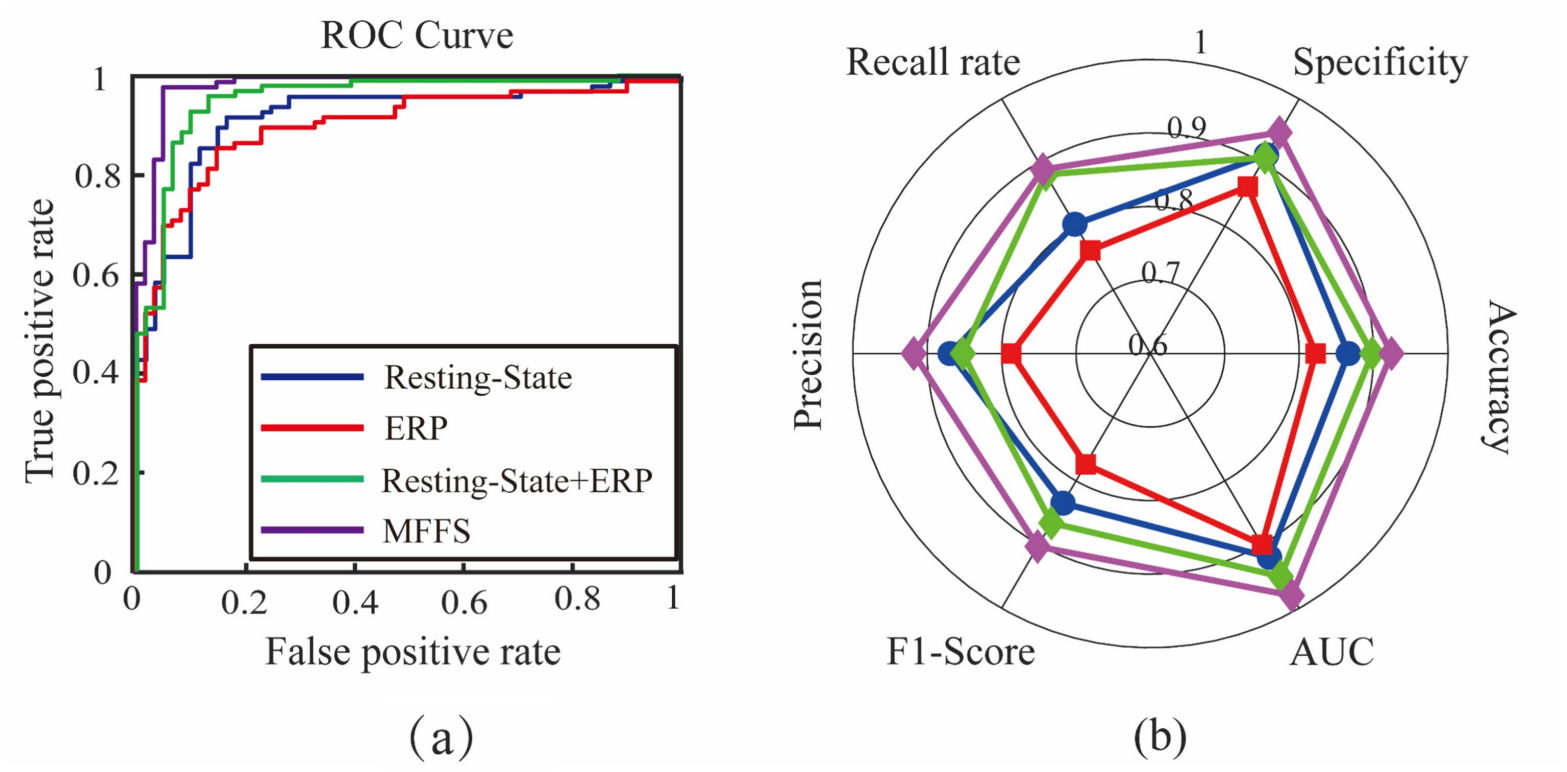
Comparison of classification performance of different feature sets: **(a)** Classification model ROC curve, **(b)** Classification indicator radar chart.

To provide a more intuitive comparison of the classification ability of different feature sets, we plotted the ROC curves and radar charts in Figure 12. In the figure, the blue line represents resting-state EEG features, the red line represents ERP features, the green line represents the combined resting-state + ERP features, and the purple line represents the MFFS-optimized feature set. The visualizations clearly show that the MFFS-optimized feature set, which fuses resting-state and auditory-evoked EEG features, achieves significantly better classification performance than either single-modal EEG features or simply combined multi-modal feature sets.

## 4 Discussion

This study conducted an evaluation of consciousness levels in DOC patients and found that: (1) The NDF of MCS patients at rest were significantly higher than those of UWS patients. Comparative analysis of SC and NI showed that SC’s ability to distinguish the two groups of patients was significantly higher than NI, which may reflect that SC is more able to reflect the overall neural network. Status and complexity; (2) Increased neuromodulation intensity (NI) in patients with MCS may be associated with higher-level consciousness networks. The difference in NI between MCS and UWS patients (reflecting the proportion of neural modulation activity) is higher in MCS than in UWS, possibly due to the significant damage to the subcortical-frontotemporal-parietal connection in UWS patients (Panda et al., 2022). Aubinet et al. (2019), found that the recovery of command-following ability was synchronized with the reconstruction of language and internal consciousness networks. The feature sets optimized in this study (such as NI-Alpha) may indirectly capture the residual activity of these networks, providing clues for exploring the potential internal consciousness of patients with DOC. (3) The ability to automatically detect auditory deviations, as reflected by MMN, is a manifestation of the integrity of the auditory pathway, just like auditory localization. Studies have found that compared with UWS patients, MCS patients exhibited significant differences in the N1 and MMN EEG characteristics induced by auditory stimulation, and the MMN amplitude in MCS patients was significantly higher than that in UWS. This is because damage to the thalamic-frontotemporal broadcasting system in DOC patients directly affects the transmission and integration of auditory information (Panda et al., 2023). Research results show MCS patients retain a more complete auditory processing network (Carrière et al., 2020); (4) The ESC and ENI values of MCS patients were higher than those of UWS patients, and the discrimination effect of ESC was higher than that of ENI; (5) using SVM algorithms to construct evaluation models for NDF and ERP features achieved average grouping accuracies of 67.5 and 74.5%, respectively. Combining the above EEG features resulted in a classification accuracy of 89.8%, and then using the MFFS algorithm optimized all EEG features, achieving an average grouping accuracy of 92.4%.

Since the participants in our study are patients with UWS and MCS, the P3 component—which depends on active cognitive participation—was not sufficiently stable. Therefore, we did not analyze the P3 component.

In our study, the proposed consciousness level evaluation method for DOC patients—combining resting-state and auditory-evoked EEG features—achieved an average classification accuracy of 92.4% in differentiating MCS and UWS patients. Compared with the grouping results reported currently, this classification method had obvious advantages. For example, Carozzo et al. achieved an accuracy of 88.6% (Porcaro et al., 2022), Sun et al. achieved an accuracy of 85.7% (Sun et al., 2023), and Li et al. achieved an accuracy of 82% (Wang et al., 2022). The possible reasons for the excellent performance of this method were as follows: First, the combination of two different modalities of brain states, resting-state and auditory-evoked stimuli, can complement each other’s information and more comprehensively reflect the brain’s consciousness state. Second, the MFFS feature selection algorithm was combined to remove redundant features after combination and avoid classifier overfitting. Third, the database used in this study contained a large number of patient samples, providing sufficient data for the classifier to train and achieve better performance.

We propose multimodal fusion combining resting and task states for the classification task of DoC patients, bridging the information gap of unimodal states. Optimization of features using the MFFS algorithm improves classification accuracy and provides a more reliable reference for clinical treatment.

When using NDF and ERP individually for classification, it was found that the average grouping accuracy of NDF was the highest. The reason for this may be that the discharge activity of brain neurons is non-stationary and nonlinear, so the EEG signal is also a nonlinear time series signal, and nonlinear features can better describe these changes. Recent research also showed that in most cases, brain signals did not represent sustained oscillations at specific frequencies, but represent intermittent repetitive (aperiodic) transient activity (Feingold et al., 2015; Lundqvist et al., 2016), so linear analysis was not suitable for describing the irregular and non-periodic patterns of EEG signals. In addition, nonlinear features contained more information in the EEG signal, such as phase synchronization and chaos, which were difficult to describe using linear methods such as power spectrum analysis. Related research results also showed that PSD performs well in distinguishing between normal subjects and patients, but it was difficult to distinguish patients with different levels of consciousness (Lehembre et al., 2012; Lehembre et al., 2012; Riganello et al., 2021). This result further confirmed that non-linear features in resting-state EEG were superior to power spectrum features when it came to fine-grained classification of DOC patients. Therefore, we do not use PSD features.

## 5 Conclusion

The consciousness assessment method proposed in this article shows a strong ability to extract and integrate multi-modal EEG features to characterize neural activities related to consciousness. This multi-modal feature extraction and integration improves the accuracy of patient classification, enabling more precise differentiated diagnosis in the grouping of patients with disorders of consciousness (DOC). By overcoming the limitations of traditional methods, the method not only enhances diagnostic reliability but also helps identify subtle neural patterns associated with different levels of consciousness. Furthermore, its potential applications extend beyond clinical assessment to provide valuable insights into rehabilitation strategies and therapeutic interventions for patients with DOC.

## Data Availability

The datasets presented in this article are not readily available because patient privacy. Requests to access the datasets should be directed to 1226sxc@gmail.com.
